# Identification of genes associated with sperm storage capacity in hens at different times after insemination by RNA-seq and Ribo-seq

**DOI:** 10.1186/s12864-024-10472-2

**Published:** 2024-06-03

**Authors:** Ruitang Chai, Cong Xiao, Zhuliang Yang, Wenya Du, Ke Lv, Jiayi Zhang, Xiurong Yang

**Affiliations:** 1 College of Animal Science and Technology, Guangxi University, Nanning, 530004 China; 2 Guangxi Key Laboratory of Animal Breeding, Disease Control and Prevention, Nanning, 530004 China

**Keywords:** Sperm storage capacity, Sperm storage tubule, RNA-seq, Ribo-Seq, Guangxi partridge chicken

## Abstract

**Background:**

Sperm storage capacity (**SSC**) determines the duration of fertility in hens and is an important reproduction trait that cannot be ignored in production. Currently, the genetic mechanism of SSC is still unclear in hens. Therefore, to explore the genetic basis of SSC, we analyzed the uterus-vagina junction (**UVJ**) of hens with different SSC at different times after insemination by RNA-seq and Ribo-seq.

**Results:**

Our results showed that 589, 596, and 527 differentially expressed genes (**DEGs**), 730, 783, and 324 differentially translated genes (**DTGs**), and 804, 625, and 467 differential translation efficiency genes (**DTEGs**) were detected on the 5th, 10th, and 15th days after insemination, respectively. In transcription levels, we found that the differences of SSC at different times after insemination were mainly reflected in the transmission of information between cells, the composition of intercellular adhesion complexes, the regulation of ion channels, the regulation of cellular physiological activities, the composition of cells, and the composition of cell membranes. In translation efficiency (**TE)** levels, the differences of SSC were mainly related to the physiological and metabolic activities in the cell, the composition of the organelle membrane, the physiological activities of oxidation, cell components, and cell growth processes. According to pathway analysis, SSC was related to neuroactive ligand-receptor interaction, histidine metabolism, and PPAR signaling pathway at the transcriptional level and glutathione metabolism, oxidative phosphorylation, calcium signaling pathway, cell adhesion molecules, galactose metabolism, and Wnt signaling pathway at the TE level. We screened candidate genes affecting SSC at transcriptional levels (*COL4A4*, *MUC6*, *MCHR2*, *TACR1*, *AVPR1A*, *COL1A1*, *HK2*, *RB1*, *VIPR2*, *HMGCS2*) and TE levels(*COL4A4*, *MUC6*, *CYCS*, *NDUFA13*, *CYTB*, *RRM2*, *CAMK4*, *HRH2*, *LCT*, *GCK*, *GALT*). Among them, *COL4A4* and *MUC6* were the key candidate genes differing in transcription, translation, and translation efficiency.

**Conclusions:**

Our study used the combined analysis of RNA-seq and Ribo-seq for the first time to investigate the SSC and reveal the physiological processes associated with SSC. The key candidate genes affecting SSC were screened, and the theoretical basis was provided for the analysis of the molecular regulation mechanism of SSC.

**Supplementary Information:**

The online version contains supplementary material available at 10.1186/s12864-024-10472-2.

## Background

With the development of poultry industry, the duration of fertility (**DF**) in hens is crucial in family-based breeding and the production of commodity generation. In poultry production, the DF refers to the ability of hens to maintain fertilization for a long period of time and continue to produce a large number of fertile eggs [1, 2]. The more fertile eggs that a hen continues to produce after artificial insemination was correlated to the higher DF and subsequent higher economic benefits of the breeding enterprise. Research has shown that after artificial insemination, sperm are temporarily stored in the sperm storage tubules (**SSTs**) in the uterus-vagina junction (**UVJ**) and then discharged over time so that hens can remain continuously fertilized [1]. The higher capacity of the SST to store sperm, the higher DF. Therefore, a great deal of research has been done to improve sperm storage capacity (**SSC**) or DF.

Studies have shown that SSC is mainly related to phenotypic differences in the hen’s UVJ [3, 4] and the environment of sperm storage provided by the SST and the UVJ [5–10]. However, the underlying causes are mainly due to differences in gene expression and molecular genetic mechanisms. Transcriptome sequencing of the UVJ in turkeys on days 1, 7, 30, 60, and 90 after insemination showed that functions such as immune response, lipid synthesis and transfer, cytoskeletal reorganization, and regulation of ions in the SSTs affect the SSC in hens [11]. Fatty acid metabolism, regulation of cell differentiation, pH regulation, transporter regulation, and immune response may be related to the SSC by transcriptome sequencing of UVJ between the high sperm storage capacity (**HSSC**) and low sperm storage capacity (**LSSC**) groups. And they screened for genes that may regulate the SSC, such as *CAIV*, *SLC4A4* [12]. Meanwhile, *HIP1* and *PDE1C* may also be candidate genes for the DF in hens [13].

For molecular biology research, quantitative information on gene expression is the most fundamental data. For gene expression quantification, the most common quantitative methods are transcriptome and proteome. RNA-seq is an important method to understanding the molecular mechanisms that underpin biological processes and phenotypes in a variety of biological contexts from the transcriptional level. However, the genetic central dogma followed by biology is a very complex process, and the expression of mRNA ultimately depends on mRNA stability, mRNA degradation and translation efficiency (**TE**). Meanwhile, the share of translational regulation inside the cell is about 54% [14], which exceeds all other regulations combined and is the main way of life regulation. By comparing the transcriptome and proteome of different species, it was found that the correlation between the expression level of mRNA and the expression of its encoded proteins was low, suggesting that the expression levels of mRNA could not be a direct measure of protein level [15]. Therefore, single RNA-seq can not capture the full view of gene expression. Combined multi-omics analyses are more conducive to revealing the complex growth and developmental regulatory mechanisms of the animal organism, and can deeply excavate key candidate genes [16, 17].

Ribosome profile sequencing (**Ribo-seq**) is a study of the translation process from RNA to protein. Ribosome-protected fragments (**RPFs**) are sequenced to accurately obtain information and precise quantification of all translatable molecules in a sample, including mRNA and other potentially translatable RNA molecules, such as lncRNA, circRNA, and so on. Ribo-seq can detect the TE of genes and study the translational regulation of the transcriptome, which is a bridge between the transcriptome and proteome [18]. Therefore, correlation analysis of RNA-seq and Ribo-seq can provide a clearer understanding of the mechanism of gene expression regulation. Previous research has utilized Ribo-seq and RNA-seq to detect more translationally regulated genes, leading to a clearer understanding of the translationally regulated mechanisms involved in the physiological processes that cause disease [19]. By using Ribo-seq and RNA-seq to investigate acute myeloid leukemia, a small tumor peptide encoded by non-coding RNA were successfully detected, which provided a theoretical basis for targeting the translational mechanism of cancer cell [20]. Yan et al. investigated the physiological process of cardiomyocyte hypertrophy and elucidated the translational mechanism of cardiomyocyte hypertrophy using Ribo-seq and RNA-seq [21].

At present, a series of studies have been conducted to explore the SSC using transcriptomics. By transcriptome sequencing of inseminated and uninseminated UVJ, Atikuzzaman et al. showed for the first time that the process of sperm entering the UVJ and being stored in the SSTs induces changes in the expression of some of the genes in the UVJ. The genes *IFIT5*, *IFI16*, *SLC16A2*, and *SLC4A9* affect the storage of sperm in the oviduct, mainly by participating in immune-regulation and pH-regulatory functions [22, 23]. By sequencing the lncRNA transcriptome of UVJs from the HSSC and LSSC groups, it was found that most of the target genes of the differential lncRNAs were enriched in molecular functions, such as the development of reproductive structures, developmental processes involved in reproduction, response to cytokines, carbohydrate binding, chromatin organization, and immune pathways [6]. Transcriptome sequencing of UVJ in hens at different ages showed there were differences in lipid anabolism and immune function, which in turn affected SSC [24]. In order to maintain the normal survival environment of sperm in the SSTs, the SSTs and UVJ will satisfy the normal storage of sperm by a series of physiological activities with the change of storage time. However, current studies cannot demonstrate the expression trends of genes related to SSC over time after insemination, and transcriptome sequencing analysis alone cannot capture the full view of gene expression. Therefore, integrating multi-omics data has become a popular method nowadays. Ribo-seq is an emerging technology to study the translational footprint by analyzing mRNA fragments bound to ribosomes, and the combined analysis of Ribo-seq and RNA-seq will also allow us to get a clearer view of how genes change at the transcriptional level and the translational level [25].

Therefore, the aim of the present study was to investigate the expression trends of UVJ genes over time after insemination by RNA-seq and Ribo-seq, to analyze the expression changes of genes at the transcriptional and translational levels, to analyze the differences in SSC at the transcriptional and translational levels, and to screen the important candidate genes and signaling pathways affecting the SSC. This study provides a theoretical basis for the analysis of the molecular regulation of SSC.

## Methods

### Sample collection

The experimental animals used in this study were Guangxi partridge chickens provided by Guangxi Hongguang Agricultural and Animal Husbandry Co., LTD. To avoid the influence of the sperm quality of roosters on the SSC of hens, we strictly selected the roosters before the beginning of the experiment. Sperm motility and concentration was subjectively assessed under 200× magnification using a portable microscope. Finally, 216 hens (age, 28 weeks) and 21 roosters (age, 35 weeks) with satisfactory semen quality were selected for the experiment. All experimental animals were housed in single cages and fed according to the company’s standard feeding protocol, with a daily light duration of 16 h. After mixing the pooled semen, it was immediately diluted with an equal volume of semen diluent (information on the composition of the semen diluent as follows: sodium glutamate, tri-potassium citrate, magnesium acetate, sodium acetate, glucose). All hens were inseminated twice consecutively with 0.03 mL of diluted semen, and the production of fertilized eggs by each hen was counted within 15 days after insemination. Fertilization rate (**FE**) and fertility duration day (**FDD**) were utilized in this experiment to reflect the level of SSC. FE was calculated as the ratio of the total number of all fertile eggs to the total number of all incubated eggs within 15 days after insemination. FDD was defined as the number of days until laying the last fertilized egg before the cumulative total of two unfertilized eggs within 15 days after insemination minus the number of days of laying an unfertilized egg [26]. Based on the two-tailed method, 9 hens with HSSC and 9 hens with LSSC were selected as subsequent experimental individuals from 216 hens. These 18 hens were randomly divided into three groups, with 3 hens of HSSC and LSSC in each group. Afterwards, the hens in these three groups were again inseminated twice consecutively, and the UVJ samples were collected on days 5, 10, and 15 after insemination, respectively. Euthanasia prior to sample collection was performed by cervical dislocation while minimizing the chicken’s suffering as much as possible. The UVJ was divided longitudinally into two parts, one for RNA-seq and the other for Ribo-seq.

### RNA extraction and sequencing

Total RNA was extracted from the UVJ tissues using TRIzol Reagent (Invitrogen, Carlsbad, CA, United States) according to the manufacturer’s instructions. RNA integrity was assessed using the Bioanalyzer 2100 system (Agilent Technologies, CA, USA). Sequencing was then performed on the Illumina noveseq 6000 platform at Novogene (Novogene, Beijing, China).

### Bioinformatics analysis of RNA-seq

Firstly, raw reads were filtered using Trimmomatic (v0.39), and the filtering included removing reads with adapter contamination, removing reads with a ratio of uncertain bases greater than 0.002, and removing single-ended reads containing low quality bases that exceeded the ratio of the length of that read by 50%. The clean data was obtained after filtering the raw sequencing data. Then, the error rate, GC content, Q20, and Q30 were counted using fastp (v0.20.1) software to ensure that the quality of the clean reads was qualified before performing the reference genome comparison. The chicken reference genome file and genome annotation file were downloaded from the Ensembl website. The reference genome version was GRCg7b, and clean reads were compared with the reference genome using Hisat2 (v2.2.1) software. The sam files obtained from the comparison were converted to sorted bam files by samtools (v1.1.4), and the count number of each gene was calculated using HTseq-count (v0.6.1p1). Finally, differential expression analysis was performed by DESeq2 R package (v1.34.0). Differentially expressed genes (**DEGs**) were screened based on *P* < 0.05 and |log2 FoldChange| ≥ 1 as criteria. Time-series analysis of gene expression using the Mfuzz R package. DEGs were analyzed for Gene Ontology (**GO**) and Kyoto Encyclopedia of Genes and Genomes (**KEGG**) enrichment using the clusterProfiler (v4.0.5) R package.

### Ribosome profiling

UVJ from different periods after insemination were treated with specific lysis buffer containing actinomycinone (50 mg/mL) to obtain lysates, and lysate concentrations were determined using a NanoDrop™ 2000 spectrophotometer. Firstly, the lysate solution was subjected to nucleic acid digestion and ribosome bounding RNA was separated from free mRNA. Enrichment of ribosome-mRNA complexes yielded RPFs. To digest RNAs other than RPFs, UVJ lysates were treated with the nonspecific ribonucleic acid endonuclease RNase I. Ribosome isolation was performed by molecular-exclusionchromatography using a MicroSpin S − 400 HR column, and the RNA samples were then treated with an rRNA removal kit to remove as much rRNA contamination from the samples as possible. The relatively short (20 ∼ 38nt) RPF fragments were then purified by PAGE. PAGE purification was performed to obtain the target RNA fragment. Subsequently, end repair was performed by adding 5’ and 3’ junctions directly at both ends, and then the fragments were reverse-transcribed into cDNA and amplified by PCR to obtain the initial library. PAGE gel screening of the target fragments was performed to obtain the library containing the target fragments [27, 28]. After library construction, the library concentration was measured by a Qubit® 2.0 fluorometer and adjusted to 1 ng/uL. The insert fragment size of the obtained library was checked using an Agilent 2100 Bioanalyzer. Finally, the exact concentration of the cDNA library was checked again using qPCR. Once the insert size and concentration of the library were the same, the samples were subjected to Illumina sequencing.

### Analysis of Ribo-Seq data

To obtain clean reads, the raw reads data were processed by removing reads with 5’ adapter, removing reads without 3’ adapter or insert sequence, removing reads with more than 10% uncertain bases, and removing reads with more than 50% nucleotides with Qphred < = 5. In addition, GC content was calculated for Q20, Q30, and clean reads, and all downstream analyses were based on high-quality clean reads. Utilizing the Bowtie software, sequences associated with rRNA and tRNA were filtered out, followed by alignment of the remaining sequences to the reference genome [29]. The reference genome version used is consistent with the RNA-seq. The SAM file was converted into BAM format using the SAMtools software, followed by quality control of the BAM file using the Ribo-TISH software [30]. According to the results of Ribo-TISH, frame distribution features, reads length distribution features, and P-sites distribution were extracted using custom R-script and plotted using ggpplot2. Finally, the mapping results at the gene level were quantified using HTSeq.

### Analysis of DTGs, DTEGs and the combination of the translatome and transcriptome

Differential translation analysis was performed using the DESeq2 R package (1.14.1). TE is the rate at which mRNAs are translated into proteins in the cell. TE is equal to the ratio of RPKM in Ribo-seq to RPKM in RNA-seq. Differential translation efficiency analysis was performed using Ribores. *P*-values were adjusted using the Benjamini and Hochberg method, which in turn controlled for false discovery rates. Differentially translated genes (**DTGs**) and differential translation efficiency genes (**DTEGs**) were identified according to | log2 (Fold change)| > 1 and *P* < 0.05 [31, 32]. GO and KEGG enrichment analysis of DTEGs using the clusterProfiler R package [33].

Combined RNA-seq and Ribo-seq data were compared for all expressed genes to detect trends in gene expression at both the transcriptional and translational levels.

### QRT-PCR analysis

Total RNA was reverse transcribed into cDNA after removing genomic gDNA according to the instructions of the Reverse Transcription Kit (TaKaRa, Dalian, China). Quantitative Real-time PCR (**QRT-PCR**) primers for differentially expressed genes were designed using the online website NCBI and Oligo7 software, and the primer sequence information is shown in Table S1. cDNA of UVJ tissue was used as a template to verify the accuracy of RNA-seq, and the data were used to calculate the relative expression by the 2^−ΔΔ^CT method. Amplification conditions and data calculation methods were referred to previous studies [24].

## Results

### Analysis of phenotypes with HSSC and LSSC groups

**Fig. 1:**
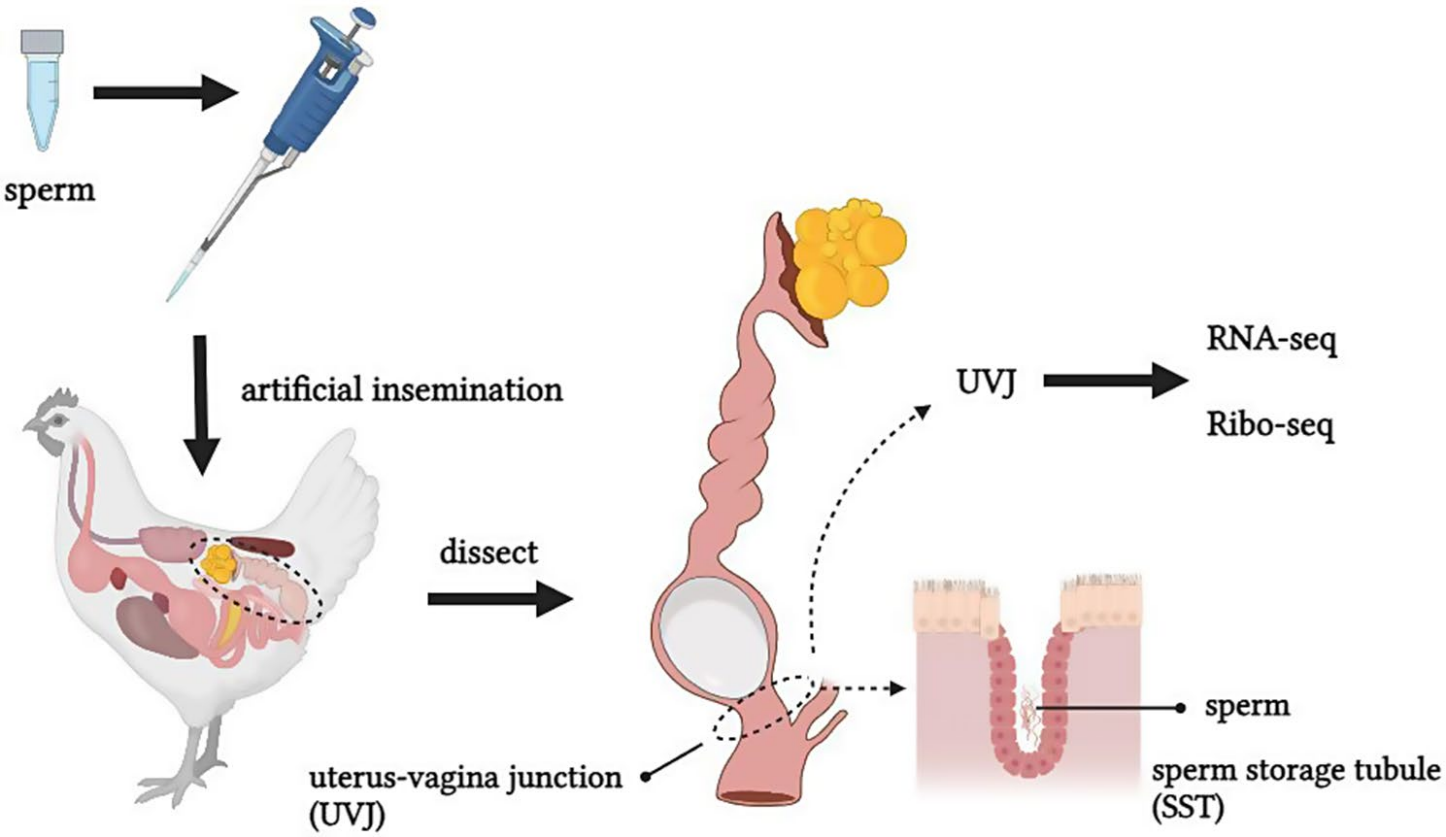
Experimental procedure

**Fig. 2:**
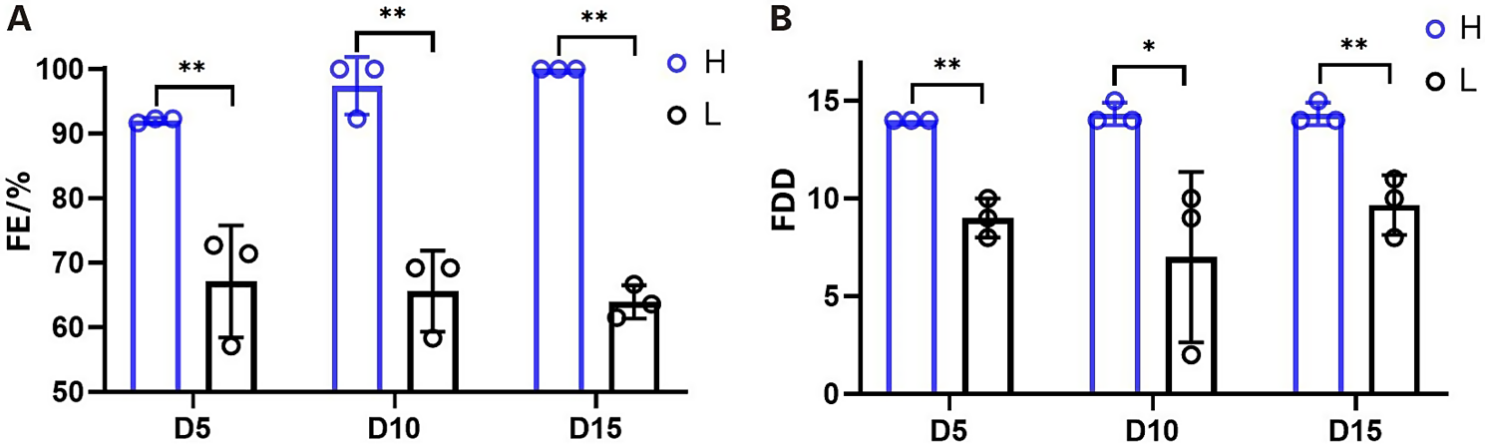
Differences in sperm storage capacity of hens in high and low sperm storage groups at different times after insemination. (**A**) FE: Fertility rate. (**B**) FDD: Fertility duration day

The experimental procedure is shown in Fig. 1. We tested the SSC of 216 hens after insemination using FE and FDD as indicators (Figure S1). Under the premise of egg production rate>80%, 9 hens each with HSSC and LSSC were selected from 216 hens by the two-tailed method as the test individuals. The results showed that the HSSC group had FE > 91% and FDD ≥ 14, whereas the LSSC group had FE < 73% and FDD ≤ 11. Differences in SSC between hens in the HSSC and LSSC groups on the 5th, 10th and 15th days after insemination were analyzed, as shown in Fig. 2. The FE of the HSSC group was significantly higher (*P* < 0.01) than that of the LSSC group in all three periods, and the FDD of the HSSC group was also significantly higher (*P* < 0.01) than that of the LSSC group on the 5th and 15th days after insemination, and only the FDD of the HSSC group was significantly higher (*P* < 0.05) than that of the LSSC group on the 10th day after insemination.

### Identification and functional enrichment analysis of differentially expressed genes

**Fig. 3:**
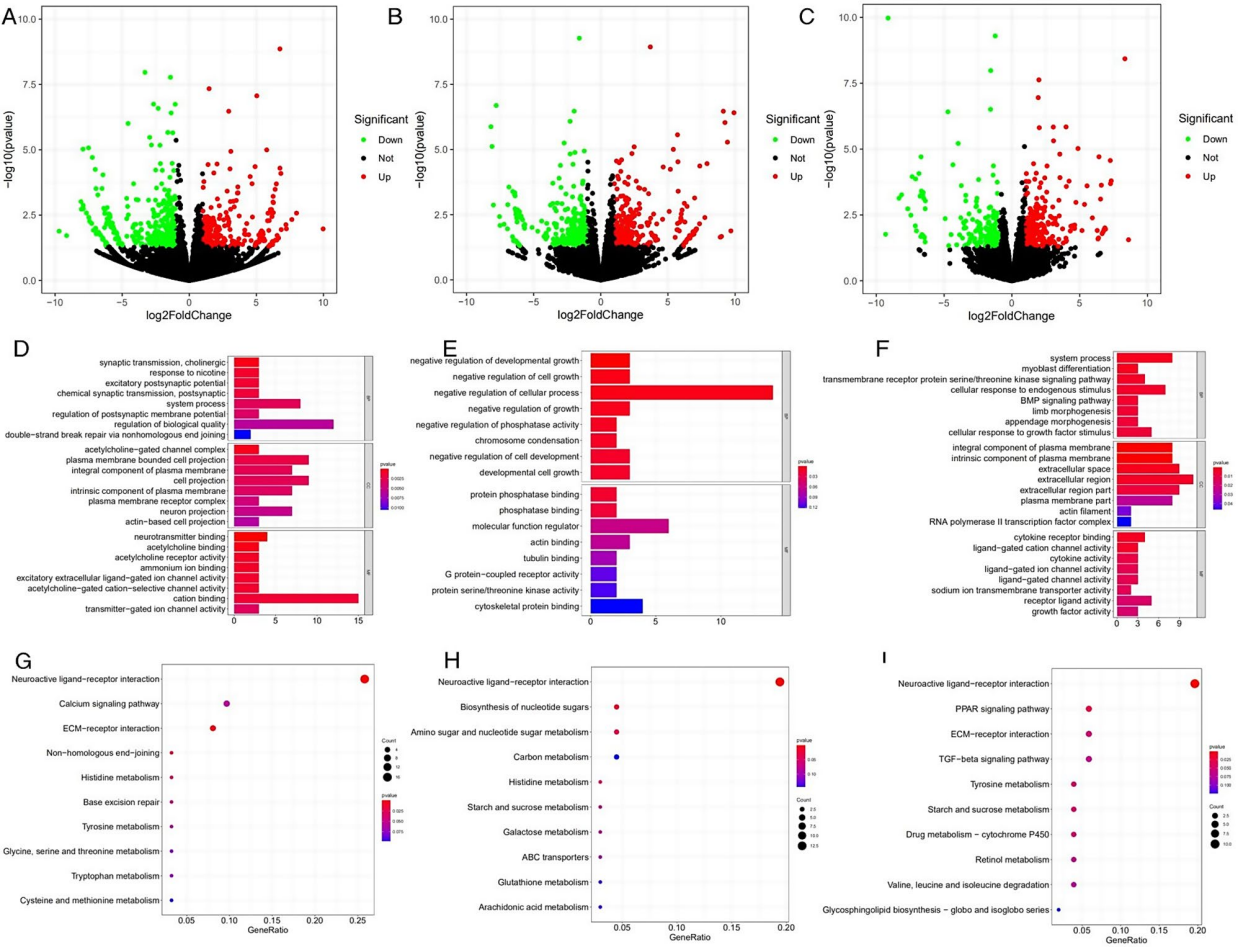
Analysis of differentially expressed genes. (**A**-**C**) Volcano plots of DEGs between UVJ in hens of HSSC and LSSC groups at different times after insemination. A, B and C represent days 5, 10, and 15 after insemination, respectively. The x-axis and y-axis represented the log2 fold change and statistical significance (-log10 of *P*-value), respectively. Transcripts with an absolute log2 fold change>1 and *P* <0.05 were set up as DEGs. The red and green dots represented the up-regulated and down-regulated DEGs, respectively. (**D**-**F**) The top GO terms in the enrichment analysis of DEGs between UVJ of HSSC and LSSC groups at different times after insemination. D, E, and F represent days 5, 10, and 15 after insemination, respectively. (**G**-**I**) The top 10 KEGG pathways in the enrichment analysis of DEGs between UVJ of HSSC and LSSC groups at different times after insemination. G, H, and I represent days 5, 10, and 15 after insemination, respectively. Bubble size represents the number of DEGs. Abbreviations: DEGs, differentially expressed genes; GO, gene ontology; KEGG, Kyoto Encyclopedia of Genes and Genomes; HSSC, high sperm storage capacity; LSSC, low sperm storage capacity

RNA-seq of 18 UVJ yielded a total of 257.45G of raw data and 252.55G of filtered clean data (Table S2). The transcriptome sequencing results of UVJ from the HSSC and LSSC groups on days 5, 10, and 15 after insemination were analyzed, and a total of 589, 596, and 527 DEGs were identified. Among them, 236, 299, and 315 were up-regulated, and 353, 297, and 212 were down-regulated (Fig. 3A and C**)**.

GO analysis of DEGs on days 5, 10, and 15 after insemination enriched 93, 57, and 100 significant terms (*P* < 0.05). Figure 3D and F listed the most significant 24, 16, and 24 terms, respectively. On day 5, the functional differences of DEGs were mainly reflected in the transmission of information between cells, the composition of intercellular adhesion complexes and the regulation of ion channels. GO terms that were highly correlated with the SSC were synaptic transmission, cholinergic, regulation of biological quality, positive regulation of MAP kinase activity, transmembrane transport, plasma membrane bounded cell projection, cation binding etc. On day 10, the functional differences of genes were mainly reflected in the regulation of cellular physiological activities and the composition of cells. GO terms that were highly correlated with the SSC were negative regulation of multicellular organismal process, negative regulation of cellular process, negative regulation of hydrolase activity, regulation of growth, regulation of lipid metabolic process, cell cycle G2/M phase transition, protein phosphatase binding etc. On day 15, functional differences of genes are mainly reflected in the transmission of information between cells, the composition of cell membranes and the regulation of ion channels. GO terms that were highly correlated with the SSC were system process, cellular response to endogenous stimulus, carbohydrate transport, regulation of transmembrane receptor protein serine/threonine kinase signaling pathway, sodium ion transmembrane transport, extracellular region etc. The results are shown in Table 1.

KEGG analysis of DEGs on days 5, 10, and 15 after insemination enriched a total of 61, 72, and 65 pathways, and Fig. 3G and I shows the top 10 pathways sorted by *P*-value. On day 5, KEGG pathways that were highly correlated with SSC were neuroactive ligand-receptor interaction, ECM-receptor interaction, histidine metabolism, calcium signaling pathway, glycine, serine and threonine metabolism, PPAR signaling pathway etc. On day 10, KEGG pathways that were highly correlated with SSC were neuroactive ligand-receptor interaction, amino sugar and nucleotide sugar metabolism, starch and sucrose metabolism, calcium signaling pathway, ECM-receptor interaction, glutathione metabolism etc. On day 15, KEGG pathways that were highly correlated with SSC were neuroactive ligand-receptor interaction, PPAR signaling pathway, starch and sucrose metabolism, ECM-receptor interaction, valine, leucine and isoleucine degradation, TGF-beta signaling pathway etc. The results are shown in Table 2.

**Table 1:** GO terms of DEGs associated with sperm storage capacity at different times after insemination

Periods	GO Terms	Count	Gene
D5	synaptic transmission, cholinergic	3	*CHRNB4,CHRNA7,CHRNA1*
D5	regulation of biological quality	12	*TPH1*,***MUC6***,*CHRNB4,CHRNA7,CHRNA1,VIL1*,
			*GLDC, NTS, SEMA3C, TERF2IP, SPP1,HOXA5*
D5	positive regulation of MAP kinase activity	2	*CHRNA7,MOS*
D5	transmembrane transport	5	*CHRNB4,CHRNA7,CHRNA1,SLC7A2,GJA3*
D5	plasma membrane bounded cell projection	9	*TPH1,MYO1A, CHRNB4,CHRNA7,CHRNA1*,
			*VIL1,ABHD13,ACTN2,OPN1LW*
D5	cation binding	15	*TPH1*,***COL1A1***,*CHRNB4,CHRNA7,CHRNA1,DNTT, VIL1*,
			*LIG4,TYRP1,GLDC, GATA4,ACTN2,MATN3,PRPS2,HPX*
D10	negative regulation of multicellular organismal process	5	*PTHLH, SEMA3C, SEMA3E, HPGDS, SOSTDC1*
D10	negative regulation of cellular process	14	*CISH, PER2,BRE, PTHLH, SERPINB10B, SEMA3C, PHACTR1*,
			*NFIL3,SEMA3E, HPGDS, MASTL, SOSTDC1,RB1,DUSP4*
D10	negative regulation of hydrolase activity	3	*SERPINB10B, PHACTR1,MASTL*
D10	regulation of growth	4	*CISH, PTHLH, SEMA3C, SEMA3E*
D10	regulation of lipid metabolic process	2	*CISH, STAR*
D10	cell cycle G2/M phase transition	2	*BRE, MASTL*
D10	protein phosphatase binding	2	*PHACTR1,MASTL*
D15	system process	8	*NGF*,***MUC6***,*CHRNA1,FSHR, PLS1,HAS2,ACTC1,OPN1LW*
D15	cellular response to endogenous stimulus	7	*WNT3A, NGF, GREM1,FSHR, BMP2,MSTN, NR5A2*
D15	carbohydrate transport	2	*SLC2A14,PLS1*
D15	regulation of transmembrane receptor protein	3	*GREM1,BMP2,MSTN*
	serine/threonine kinase signaling pathway		
D15	sodium ion transmembrane transport	2	*ASIC1,SCNN1A*
D15	extracellular region	11	*WNT3A, GAL, NGF*,***MUC6***,*GREM1,SLC2A14*,
			COL17A1,BMP2,MSTN, TXLNB, HPX

**Table 2:** KEGG pathways of DEGs associated with sperm storage capacity at different times after insemination

Periods	KEGG	Count	Gene
D5	Neuroactive ligand-receptor interaction	16	*GALR2,GHRHR, GRM4,CHRNB4,CHRNA7,CHRNA1*,***TACR1***,*GRM7*,
			*GRIK3,MCHR2,UTS2B, AVPR1A, NTS, GABRB2,PLG, HTR2A*
D5	ECM-receptor interaction	5	*LAMB3*,***COL1A1***,*SV2B, LAMC2,SPP1*
D5	Histidine metabolism	2	*HNMT, CNDP1*
D5	Calcium signaling pathway	6	*CAMK1G, CHRNA7,TACR1,AVPR1A, SLC8A2,HTR2A*
D5	Glycine, serine and threonine metabolism	2	*PHGDH, GLDC*
D5	PPAR signaling pathway	2	*PLIN1,MMP1*
D10	Neuroactive ligand-receptor interaction	13	*SSTR2,PTH2R, HTR5A, ADM*,***TACR1***,***MCHR2***,*NPY2R*,
			*CHRM3,PLG, ADM2,TSHR, MLNR, P2RY14*
D10	Amino sugar and nucleotide sugar metabolism	3	*CHIA*,***HK2***,*GCK*
D10	Starch and sucrose metabolism	2	***HK2***,*GCK*
D10	Calcium signaling pathway	5	*FGF22,HTR5A*,***TACR1***,*HGF, CHRM3*
D10	ECM-receptor interaction	2	***COL4A4***,*ITGA11*
D10	Glutathione metabolism	2	*CHAC1,HPGDS*
D15	Neuroactive ligand-receptor interaction	10	*ADCYAP1R1,S1PR4,GAL*,***VIPR2***,*CHRNA1*,
			*FSHR, ADRA1D, MCHR2,NPY1R, GRM8*
D15	PPAR signaling pathway	3	*APOA5*,***HMGCS2***,*CYP7A1*
D15	Starch and sucrose metabolism	2	***HK2***,*GYG2*
D15	ECM-receptor interaction	3	*COMP, LAMB3,THBS4*
D15	Valine, leucine and isoleucine degradation	2	*AOX2*,***HMGCS2***
D15	TGF-beta signaling pathway	3	*GREM1,BMP2,ID1*

### Time-series analysis of gene expression

**Fig. 4:**
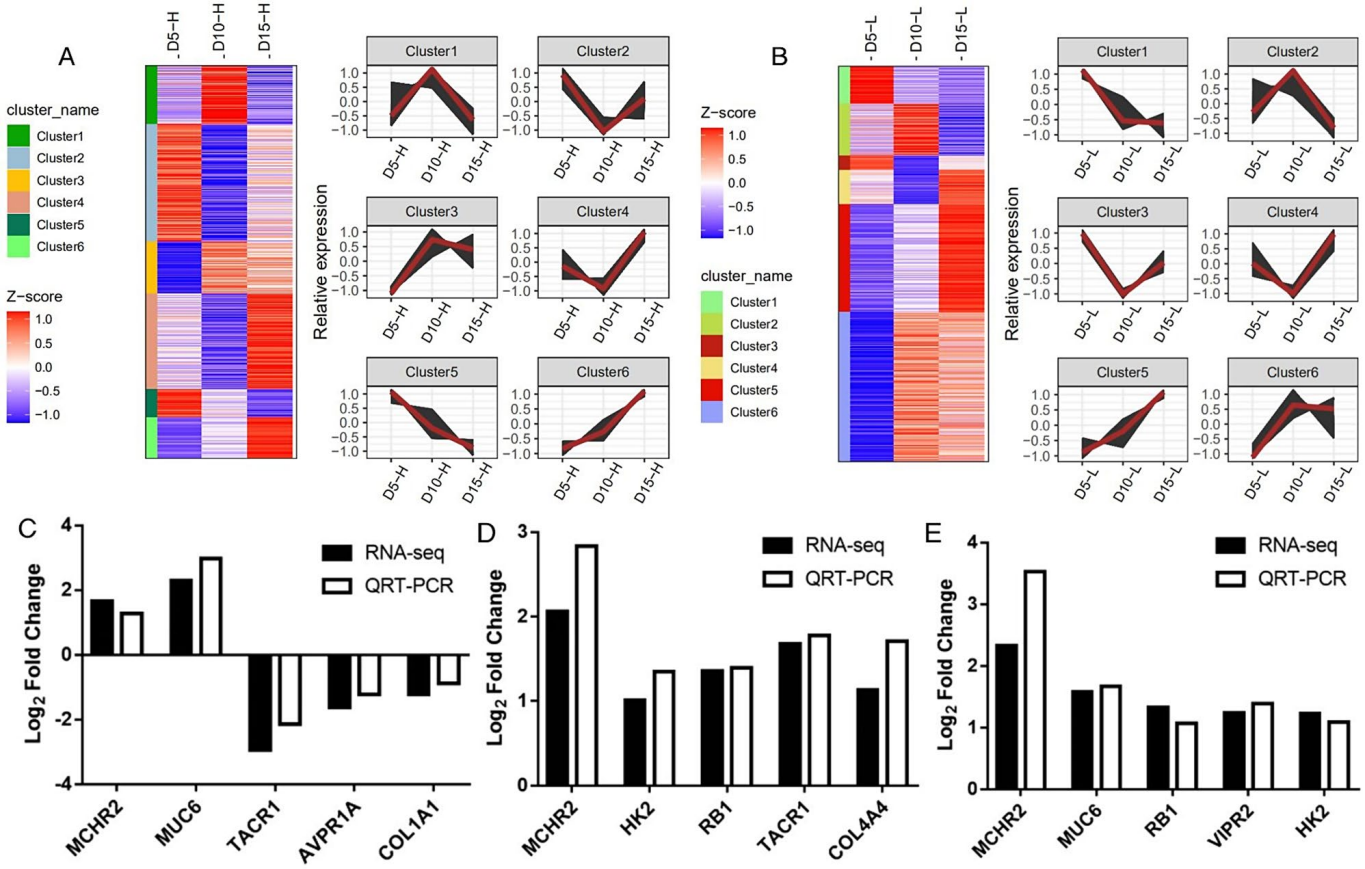
Time-Series analysis of gene expression, and QRT-PCR validation of differentially expressed genes. (**A-B**) Expression trends of all genes identified in the UVJ from day 5 to day 15 after insemination. A and B represent the HSSC and LSSC groups, respectively. (**C**-**E**) QRT-PCR validation of DEGs on days 5, 10, and 15 after insemination. C, D, and E represent days 5, 10, and 15 after insemination, respectively. Abbreviations: DEGs, differentially expressed genes; HSSC, high sperm storage capacity; LSSC, low sperm storage capacity

Time-series analysis of gene expression identified in UVJ from day 5 to day 15 after insemination was performed, and the results are shown in Fig. 4A and B. After insemination, SSC will gradually decrease over time. Therefore, the expression of genes related to SSC will gradually decrease or increase, and cluster 5, 6 in the HSSC group and cluster 1, 5 in the LSSC group were more in line with the expression trend of genes related to SSC. Based on the results of GO and KEGG pathway enrichment analysis of the DEGs, combined with the expression patterns of the genes at different times, ten candidate genes related to the SSC were screened, including *COL4A4*, *MUC6*, *MCHR2*, *TACR1*, *AVPR1A*, *COL1A1*, *HK2*, *RB1*, *VIPR2*, and *HMGCS2*. Among them, *HK2*, *VIPR2*, and *HMGCS2* was selected from cluster 6 in the HSSC group. *COL1A1* and *TACR1* was selected from cluster 1 and *COL4A4* was selected from cluster 5 in the LSSC group. Although *MCHR2*, *RB1*, and *AVPR1A* were selected from cluster 3 in the HSSC group, their expression trend in the HSSC group was still gradually increased over time. Although *MUC6* was selected from cluster 6 in the LSSC group, its expression trend in the LSSC group still gradually decreased over time.

### QRT-PCR validation of differentially expressed genes

In order to verify the accuracy of RNA-seq, 5 DEGs were selected from each period for QRT-PCR verification. Of these DEGs, nine were candidate genes for SSC, including *MCHR2*, *TACR1*, *AVPR1A*, *COL1A1*, *HK2*, *RB1*, *VIPR2*, *COL4A4*, and *MUC6*. The results were shown in Fig. 4C and E, and the expression of all genes in QRT-PCR was consistent with that in RNA-seq.

### Characterization of Ribo-Seq data and identification of differentially translated genes

**Fig. 5:**
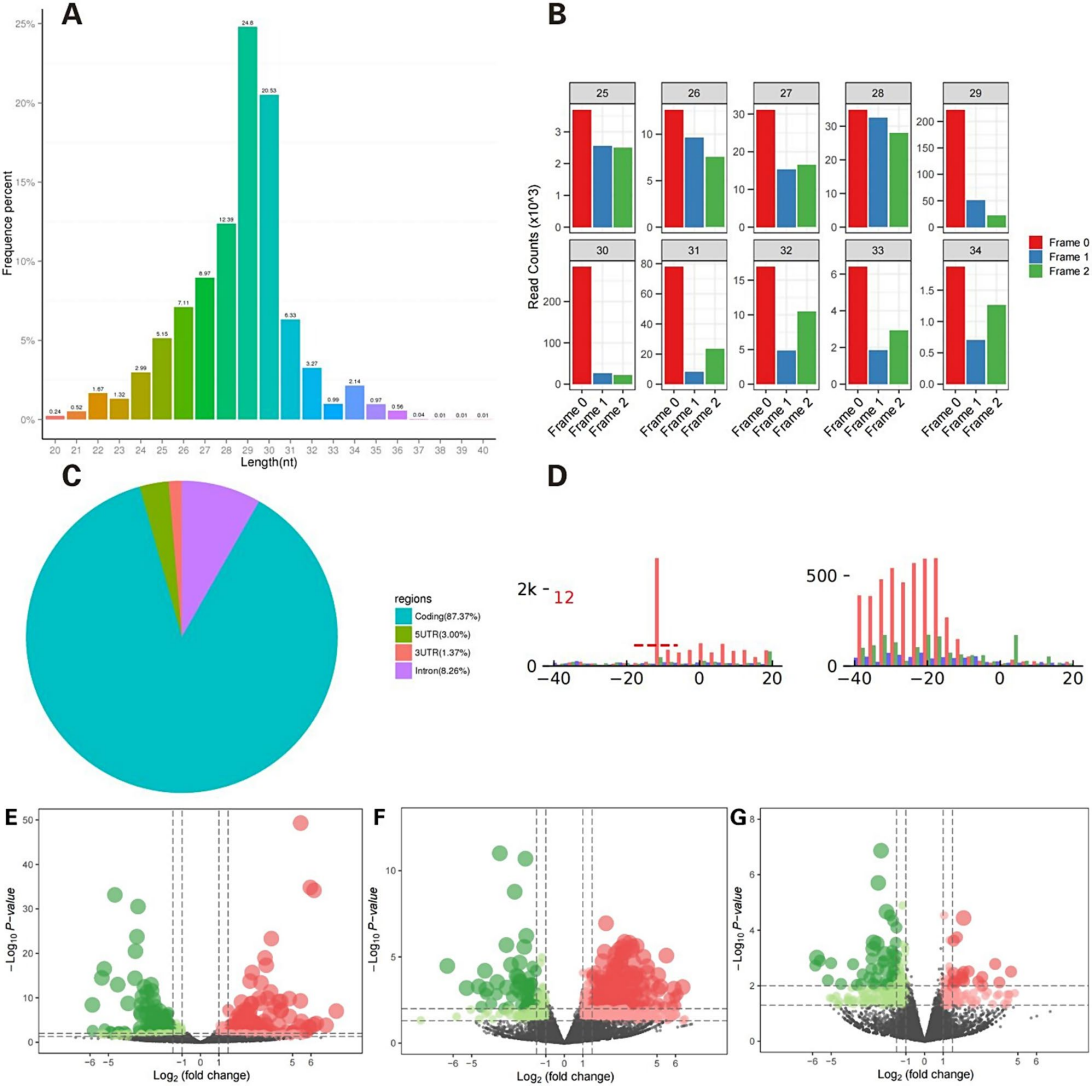
Characterization of Ribo-seq data and identification of differentially translated genes. (**A**) Statistical graph of the length of RPFs. (**B**) Distribution map of RPFs on coding genes. According to the position of RPFs on coding genes, RPFs were categorized into four groups: CDS, 5’UTR, 3’UTR, and Intron. Generally speaking, RPFs were mostly distributed in the CDS region, while their number was less in the UTR region. (**C**) Codon distribution of RPFs of various lengths (Frames 0, 1, and 2 correspond to codon bases 1, 2, and 3, respectively). Ribosomes produce stops every 3 bases (i.e., 1 codon) to complete the peptide extension of an amino acid during the sliding translation of proteins in the transcript. Since the ribosome stays longest at the first base position of the codon, the ratio of RPFs to positions corresponding to the first base of the codon is usually the highest. (**D**) Diagram of three-base rhythm analysis (start codon position on the left and position of the stop codon on the right). The depth distribution of RPFs in the CDS region was counted according to the position of the 5’ end of RPFs in the genome. Normal RPFs show a clear “high-low-low” three-base cycle pattern, whereas normal RNA-seq reads are randomly distributed across the coding genes. (**E**-**G**) Volcano plots of DTGs on days 5, 10, and 15 after insemination. E, F, and G represent days 5, 10, and 15 after insemination, respectively. The x-axis and y-axis represented the log2 fold change and statistical significance (-log10 of *P*-value), respectively. DTGs were identified according absolute log2 fold change>1 and *P* <0.05. The red and green dots represented the up-regulated and down-regulated DTGs, respectively. Abbreviations: RPFs, ribosome-protected fragments; DTGs, differentially translated genes

The raw reads obtained from Ribo-seq of 18 UVJ tissues were processed to obtain clean reads (Table S3-S4). By counting the length distribution of reads, the length of RPFs was detected to be mainly distributed in 25 ∼ 32 bp, and the data of these RPFs with the expected length will be used for the subsequent analysis, and the results are shown in Fig. 5A. The position distribution of RPFs on coding genes was counted, the coding region accounted for 87.37% of RPFs (Fig. 5B). The RPFs that were compared to the CDS region were grouped into three categories (codon 1st to 3rd bases) according to the position of the codon corresponding to the comparison position, and the proportion of the three categories of RPFs in each gene was calculated. As shown in Fig. 5C, the RPFs have the highest proportion of the first base of the codon corresponding to the pairing position. Figure 5D shows the distribution abundance of all RPFs around the start and stop codons, showing a clear “high - low - low” three-base cycle pattern.

Ribo-seq identified 730, 783, and 324 DTGs for three periods, respectively. Among them, 413, 587, and 117 were up-regulated and 317, 196, and 207 were down-regulated in translation, and the results are shown in Fig. 5E and G.

### Identification and functional enrichment analysis of differential translation efficiency genes

**Fig. 6:**
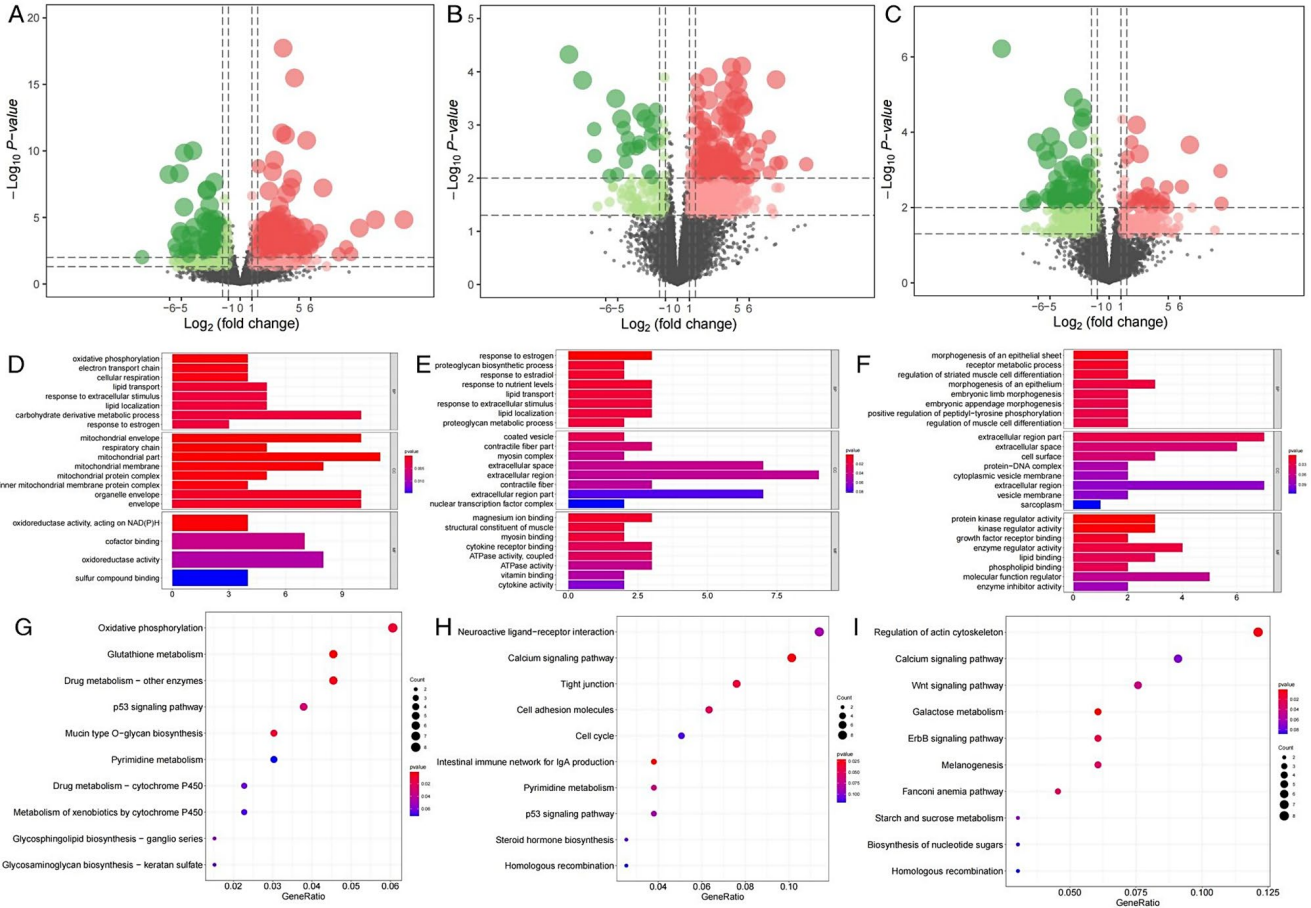
Analysis of differential translation efficiency genes. (**A**-**C**) Volcano plots of DTEGs between UVJ of HSSC and LSSC groups at different times after insemination. A, B and C represent days 5, 10 and 15 after insemination, respectively. The x-axis and y-axis represented the log2 fold change and statistical significance (-log10 of *P*-value), respectively. DTEGs were identified according absolute log2 fold change>1 and *P* <0.05. The red and green dots represented the up-regulated and down-regulated DTEGs, respectively. (**D**-**F**) The top GO terms in the enrichment analysis of DTEGs between UVJ of HSSC and LSSC groups at different times after insemination. D, E and F represent days 5, 10 and 15 after insemination, respectively. (**G**-**I**) The top 10 KEGG pathways in the enrichment analysis of DTEGs between UVJ of HSSC and LSSC groups at different times after insemination. G, H and I represent days 5, 10 and 15 after insemination, respectively. Bubble size represents the number of DTEGs. Abbreviations: DTEGs, differential translation efficiency genes; GO, gene ontology; KEGG, Kyoto Encyclopedia of Genes and Genomes; HSSC, high sperm storage capacity; LSSC, low sperm storage capacity

Ribo-seq and RNA-seq results of high and low SSC UVJ at different periods after insemination were jointly analyzed to calculate the TE of the genes and to identify DTEGs. A total of 804, 625, and 467 DTEGs were identified on days 5, 10, and 15 after insemination. Among them, 495, 513, and 145 were up-regulated for TE, and 309, 112, and 322 were down-regulated for TE, as shown in Fig. 6A and C.

GO analysis showed that DTEGs on days 5, 10, and 15 after insemination was enriched in 98, 868 and 810 terms, of which 72, 31, and 31 terms were significantly enriched (*P* < 0.05). Figure 6D and F showed the most significant 20, 24, and 24 terms, respectively. On day 5 after insemination, the functional differences of DTEGs were mainly related to the physiological and metabolic activities in the cell, the composition of the organelle membrane, and the physiological activities of oxidation. The GO terms that were highly correlated with the SSC were oxidative phosphorylation, lipid transport, carbohydrate derivative metabolic process, ATP metabolic process, small molecule metabolic process, mitochondrial envelope, oxidoreductase complex etc. On day 10, the functional differences of genes were mainly reflected in the physiological metabolic activities and the composition of cells. The GO terms that were highly correlated with the SSC were lipid transport, cell differentiation, response to lipid, magnesium ion binding, ATPase activity, coupled, extracellular space etc. On day 15, the functional differences of genes were mainly reflected in the physiological and metabolic activities of cells, cell components, and cell growth processes. The GO terms that were highly correlated with the SSC were receptor metabolic process, positive regulation of peptidyl-tyrosine phosphorylation, morphogenesis of an epithelium, regulation of phosphate metabolic process, extracellular region part, lipid binding, protein kinase regulator activity etc. The results are shown in Table 3.

KEGG pathway enrichment analysis showed that DTEGs on days 5, 10, and 15 after insemination was enriched in 12, 62, and 77 pathways, and Fig. 6G and I shows the top 10 pathways sorted by *P*-value. On day 5 after insemination, the KEGG pathways that were highly correlated with SSC were mainly glutathione metabolism and oxidative phosphorylation. On day 10, the KEGG pathways that were highly correlated with SSC were mainly intestinal immune network for IgA production, calcium signaling pathway, tight junction, cell adhesion molecules, neuroactive ligand-receptor interaction, ECM-receptor interaction etc. On day 15, the KEGG pathways that were highly correlated with SSC were mainly galactose metabolism, regulation of actin cytoskeleton, and calcium signaling pathway etc. The results are shown in Table 4.

Candidate genes related to SSC were screened based on GO and KEGG enrichment analysis of DTEGs, including *COL4A4*, *MUC6*, *CYCS*, *NDUFA13*, *CYTB*, *RRM2*, *CAMK4*, *HRH2*, *LCT*, *GCK*, and *GALT* genes.

**Table 3:** GO terms of DTEGs associated with sperm storage capacity at different times after insemination

Periods	GO Terms	Count	Gene
D5	oxidative phosphorylation	4	***CYCS***,***CYTB***,*ND4,ND4L*
D5	lipid transport	5	***MUC6***,*VTG3,RBP4A, STAR, TMEM30A*
D5	carbohydrate derivative metabolic process	10	*ST6GALNAC1,TK1,RFNG, CHST10,LYZ*,
			*CYCS, CYTB, ND4,ND4L, ATP6*
D5	ATP metabolic process	5	***CYCS***,***CYTB***,*ND4,ND4L, ATP6*
D5	small molecule metabolic process	12	*ADORA2B, TK1,DCXR, MMACHC, RBP4A, PGP*,
			*STAR, CYCS, CYTB, ND4,ND4L, ATP6*
D5	mitochondrial envelope	10	*ERAL1,BOK, STAR, SLC25A46*,***CYCS***,
			*ND6,CYTB, ND4,ND4L, ATP6*
D5	oxidoreductase complex	3	***CYTB***,*ND4,ND4L*
D10	lipid transport	3	*VTG1,VTG2,RBP4A*
D10	cell differentiation	11	*BGLAP, MUSTN1,CNTF, MYL6,CD28,MYH11*,
			*RBP4A, BMP2,CCK, LECT2,SOX21*
D10	response to lipid	4	*VTG1,VTG2,RBP4A, ALB*
D10	magnesium ion binding	3	*MYL6,FEN1,MYH11*
D10	ATPase activity, coupled	3	*MYL6,MYH11,RAD54B*
D10	extracellular space	7	*CNTF, RBP4A, BMP2,CCK, LECT2,ALB, APOV1*
D15	receptor metabolic process	2	*GREM1,NSG1*
D15	positive regulation of peptidyl-tyrosine phosphorylation	2	*GREM1,HBEGF*
D15	morphogenesis of an epithelium	3	*WNT2B, GREM1,HBEGF*
D15	regulation of phosphate metabolic process	5	*RIMBP2,GREM1,HBEGF, CCNA2,TESC*
D15	extracellular region part	7	*WNT2B, CATH3,CETP, GREM1,P3H2,HBEGF, APOV1*
D15	lipid binding	3	*CATH3,RAG2,CETP*
D15	protein kinase regulator activity	3	*GREM1,CCNA2,TESC*

**Table 4:** KEGG pathways of DTEGs associated with sperm storage capacity at different times after insemination

Periods	KEGG	Count	Gene
D5	Glutathione metabolism	6	*GSTM2,GSTT1L, NAT8B, GGCT, MGST1*,***RRM2***
D5	Oxidative phosphorylation	8	*COX10*,***NDUFA13***,***CYCS***,*ND6*,***CYTB***,*ND4,ND4L, ATP6*
D10	Intestinal immune network for IgA production	3	*CD28,ICOS, TNFRSF13B*
D10	Calcium signaling pathway	8	*GRIN1,CAMK1G, PGR2,3,CASQ2,GRIN2A, HRH2*,***CAMK4***,*FGF9*
D10	Tight junction	6	*ARPC5L, MYL6,CLDN5,MYH11,SYNPO, IGSF5*
D10	Cell adhesion molecules	5	*NTNG2,CLDN5,CD28,ICOS, CNTNAP2*
D10	Neuroactive ligand-receptor interaction	9	*GRIN1,PGR2,3,GRIN2A, P2RY4,CCK, NPY2R*,***HRH2***,*RXFP1,PLG*
D10	ECM-receptor interaction	3	*COL4A3*,***COL4A4***,*GP5*
D15	Galactose metabolism	4	*G6PC2*,***LCT***,***GCK***,***GALT***
D15	Regulation of actin cytoskeleton	8	*APC2,INSRR, ITGA2B, FGF19,F2,LPAR5,MRAS, EGF*
D15	Calcium signaling pathway	6	*GRIN2C, FGF19,HTR2B, ADCY3,PTK2B, EGF*

### Co-analysis of genes at the transcriptomics and translatomics

**Fig. 7:**
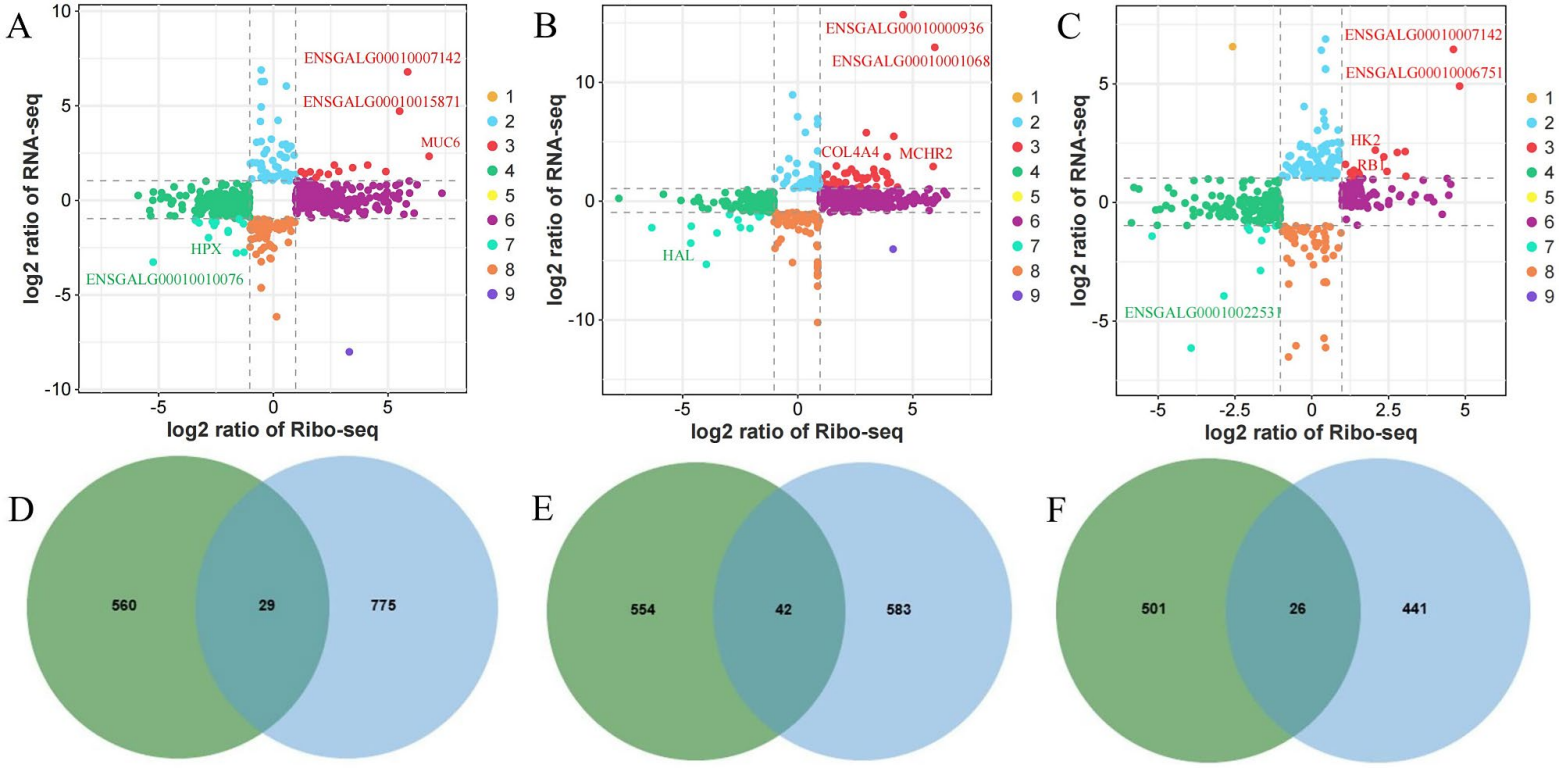
The relationship between genes at the level of transcription, translation and translation efficiency. (**A**-**C**) A, B, and C represent the expression trends of genes at the transcriptional and translational levels on days 5, 10, and 15 after insemination, respectively. Quadrants 1 and 9 indicate that the genes were differentially expressed at both transcriptional and translational levels, but with opposite expression trends. Quadrants 2 and 8 indicate that genes are differentially expressed only at the transcriptional level, but not at the translational level. Quadrants 3 and 7 indicate that the gene is differentially expressed at both the transcriptional and translational levels with the same expression trend. Quadrants 4 and 6 indicate that the gene is differentially expressed only at the translational level and not at the transcriptional level. Quadrant 5 indicates that the gene is not differentially expressed at both the transcriptional and translational levels. (**D**-**F**) D, E, and F represent the relationship between differential genes at the level of transcriptional and translation efficiency on days 5, 10, and 15 after insemination, respectively. The green and blue represented the DEGs and DTEGs, respectively. Abbreviations: DTEGs, differential translation efficiency genes; DEGs, differentially expressed genes

Ribo-seq and RNA-seq results were jointly analyzed to detect changes in gene expression during transcription and translation processes. As shown in Fig. 7A and C, on the 5th, 10th, and 15th days after insemination, there were 560, 542, and 503 genes differentially expressed only at the transcriptional level; there were 701, 729, and 300 genes differentially expressed only at the translational level; there were 28(such as *MUC6*, *HPX*), 46(such as *COL4A4*, *MCHR2*, *HAL*), and 22(such as *HK2*, *RB1*) genes differentially expressed at both transcriptional and translational levels, and the expression trends were the same. Figure 7D and F show the relationship between DEGs and DTEGs at different times after insemination. On days 5, 10, and 15 after insemination, there were 29, 42, and 26 genes that were differentially expressed at both the transcriptional level and translation efficiency level, respectively. Combined the comparative analysis of genes at the transcription, translation and TE level, the key candidate genes *MUC6* and *COL4A4* that were significantly different in HSSC and LSSC groups and had the same expression trends in three levels were screened out.

## Discussion

The KEGG analysis showed that the SSC was associated with pathways such as neuroactive ligand-receptor interaction, ECM receptor interaction, calcium signaling pathway, starch and sucrose metabolism, and PPAR signaling pathway. Similarly, it has been suggested that SSC in hens may be associated with lipid metabolism and calcium signaling pathway [5]. Transcriptome sequencing of UVJ at different times after insemination showed that the biosynthetic pathway of glycosaminoglycans, calcium signaling pathway may be related to the duration of SS in the SSTs [13], which is consistent with the signaling pathways related to SSC in this experiment: calcium signaling pathway, starch and sucrose metabolism, and PPAR signaling pathway.

Among the candidate genes *COL4A4*, *MUC6*, *MCHR2*, *TACR1*, *AVPR1A*, *COL1A1*, *HK2*, *RB1*, *VIPR2*, and *HMGCS2* screened at the transcriptional level, *MCHR2* was the only gene differentially expressed in all 3 periods after insemination. Studies have shown that MCH promotes the synthesis and secretion of the adipocyte-specific secretory factor Leptin, which in turn promotes adipocyte differentiation and lipid metabolism [34–37]. MCHR2, as a receptor for MCH, also has the potential to regulate lipid metabolism. It is hypothesized that *MCHR2* may affect the SSC in hens by participating in the lipid metabolic process in UVJ. *AVPR1A* is associated with the ion transport mechanism of male vas deferens epithelial cells [38]. Since the vas deferens and the SST also serve as sites for sperm storage, *AVPR1A* may also regulate the ion transport mechanism of SST epithelial cells to influence SSC. *VIPR2* regulates the immune function of immune T cells in mice [39], and sperm storage is also affected by immune function when stored in the UVJ [12]. Therefore, it is hypothesized that *VIPR2* also affects the immune function of UVJ, which in turn affects SSC. In addition, a series of other differential genes were identified that may influence SSC. For example, studies on human sperm function have shown that *TACR1* and *HK2* are associated with the regulation of sperm viability [40, 41]. *COL1A1* is associated with the process of sperm capacitation in the sheep epididymis, which is the site of sperm storage in sheep and has the same function as SST [42]. It has been shown that *RB1* affects sperm integrity in mice and thus regulates sperm viability [43]. Sperm survival in the SST requires sugars for energy, and *HMGCS2* is able to inhibit energy provision by inhibiting the process of glycolysis [44]. These studies suggest that *TACR1*, *HK2*, *COL1A1*, *RB1*, and *HMGCS2* may affect the SSC by regulating the viability and activity of sperm stored in the SSTs.

Candidate genes related to SSC were also screened based on GO and KEGG analysis of DTEGs, including *COL4A4*, *MUC6*, *CYCS*, *NDUFA13*, *CYTB*, *RRM2*, *CAMK4*, *HRH2*, *LCT*, *GCK*, and *GALT*. Although the genes *CYCS*, *NDUFA13*, *CYTB*, *RRM2*, *CAMK4*, *HRH2*, *LCT*, *GCK*, and *GALT* are not differentially expressed at the transcriptional level, the TE during translation is differential. And the final expression at the protein level is possibly to be differentially expressed. Therefore, these genes are also worth going over. Among them, *CYCS*, *NDUFA13*, and *CYTB* were selected from the oxidative phosphorylation signaling pathway. Oxidative phosphorylation is one of the important ways for spermatozoa to gain energy [45]. Therefore, *CYCS*, *NDUFA13*, and *CYTB* may affect the SSC by regulating the sperm viability stored in the SST. *RRM2* is selected from the glutathione metabolic pathway. Glutathione metabolic process has antioxidant function and can reduce the damage of membrane structure [46]. Therefore, it is possible that *RRM2* affects sperm membrane integrity, which in turn affects sperm viability stored in the SST and regulates SSC. *CAMK4* and *HRH2* are selected from the calcium signaling pathway. SSTs secrete calcium ions to regulate the internal environment of the SSTs [47], and it is hypothesized that *CAMK4* and *HRH2* regulate the internal environment of the SST lumen, and thus sperm motility, through the calcium signaling pathway. The galactose metabolism pathway is closely related to sperm motility [41], and genes such as *LCT*, *GCK*, and *GALT*, selected from the galactose metabolism pathway, are also candidate genes that deserve to be scrutinized.

Combined RNA-seq and Ribo-seq were analyzed and finally screened to obtain *MUC6* and *COL4A4* that were differentially expressed at the transcriptional level, translational level and TE level with the same expression trend as the key candidate genes for the SSC of hens. They are both up-regulated genes differentially expressed at the transcriptional level, while translational regulation further deepens this difference in expression. MUC6 is a type of secreted mucin. It was first found to be specifically expressed in the glands of the human gastric mucosa, and the secreted mucus plays a role in lubricating and protecting the gastric mucosa [48]. Similarly, *MUC6* was expressed in the UVJ mucosa of oviduct in hens. It has been shown that mucins can act as adhesion and anti-adhesion molecules in cell invasion, migration and intracellular signaling [49]. It is hypothesized that *MUC6* affects sperm stored in the SST by regulating the epithelial cells of the SST in the UVJ. *COL4A4* is selected from the ECM-receptor interaction pathway. The extracellular matrix (ECM) is a complex architecture of macromolecules secreted by cells to the outside of the cell, connecting tissue structures, regulating the function of tissues, and influencing the physiological activities of cells [50]. The ECM-receptor-interaction pathway is associated with the mechanism by which epithelial cells in the epididymis regulate sperm maturation [42]. Sperm in the SST is also regulated by SST epithelial cells, so it is possible that *COL4A4* affects the physiological activity of SST epithelial cells through the ECM-receptor-interaction pathway, which in turn affects SSC.

Although there are some researches on the genetic mechanism of SSC, they only stay in the use of omics sequencing to explore the genetic mechanism, and there is no relevant functional verification at the cell or tissue level. Therefore, future research should focus on in-depth studies of the molecular mechanisms and gene expression patterns within the SST. We will make full use of the candidate genes screened by omics sequencing analysis to carry out research on the molecular regulation of SSC, and explore its function and regulatory mechanism at a deeper level.

## Conclusions

This study investigated the gene expression of UVJ with different SSC at different periods after insemination by using combined RNA-seq and Ribo-seq analysis at first time. We found that the SSC was mainly associated with signaling pathways such as neuroactive ligand-receptor interaction, ECM receptor interaction, calcium signaling pathway, starch and sucrose metabolism, and PPAR signaling pathway. The genes (*COL4A4*, *MUC6*, *MCHR2*, *TACR1*, *AVPR1A*, *COL1A1*, *HK2*, *RB1*, *VIPR2*, *HMGCS2*) identified from the transcriptional level, and the genes (*MUC6*, *COL4A4*, *CYCS*, *NDUFA13*, *CYTB*, *RRM2*, *CAMK4*, *HRH2*, *LCT*, *GCK*, *GALT*) identified from the translational level were screened as candidate genes that may affect the SSC in hens. Among them, *MUC6* and *COL4A4* genes, which were differentially expressed at the transcriptional level, translational level and translation efficiency level, might be the key candidate genes affecting SSC. This study reveals the physiological processes associated with SSC and provides a theoretical basis for analyzing the molecular regulation of SSC.

### Electronic supplementary material

Below is the link to the electronic supplementary material.

Supplementary Material 1

Supplementary Material 2

Supplementary Material 3

Supplementary Material 4

Supplementary Material 5

## Data Availability

The RNA and Ribo sequencing data used and analyzed during the current study are available from the NCBI (accession number: PRJNA1113713).

## Abbreviations

DF: Duration of fertility
SSTs: Sperm storage tubules
UVJ: Uterus-vagina junction
SSC: Sperm storage capacity
HSSC: High sperm storage capacity
LSSC: Low sperm storage capacity
FE: Fertilization rate
FDD: Fertility duration day
GO: Gene Ontology
KEEG: Kyoto Encyclopedia of Genes and Genomes
DEGs: Differentially expressed genes
DTGs: Differentially translated genes
DTEGs: Differential translation efficiency genes
TE: Translation efficiency
RPFs: Ribosome-protected fragments
Ribo-seq: Ribosome profile sequencing
QRT-PCR: Quantitative Real-time PCR
