# Supplementary material for: Identification of genes associated with sperm storage capacity in hens at different times after insemination by RNA-seq and Ribo-seq
Source: BMC Genomics. 2024 Jun 3;25:554. doi: 10.1186/s12864-024-10472-2 (PMC11145833; doi:10.1186/s12864-024-10472-2)

**Supplementary file 5: Figure S1.** The statistical results of laying rate, FE and FDD within 15 days after insemination.


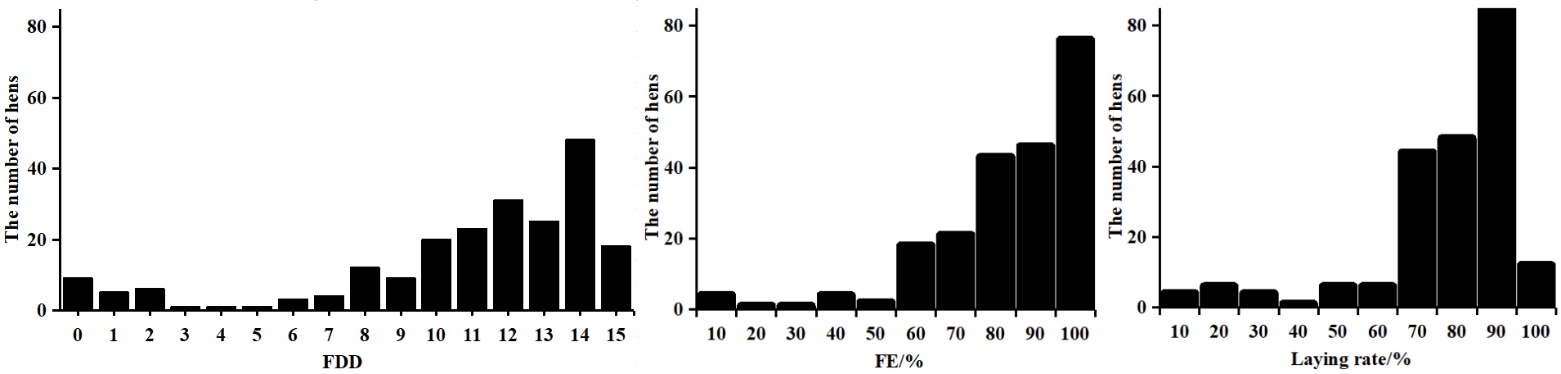

Supplement: Supplementary file 5 — Supplementary Material 5 [file 12864_2024_10472_MOESM5_ESM.docx]
